# A Risk-Based Framework for Assessing the Effectiveness of Stratospheric Aerosol Geoengineering

**DOI:** 10.1371/journal.pone.0088849

**Published:** 2014-02-12

**Authors:** Angus J. Ferraro, Andrew J. Charlton-Perez, Eleanor J. Highwood

**Affiliations:** Department of Meteorology, University of Reading, Reading, United Kingdom; Plymouth University, United Kingdom

## Abstract

Geoengineering by stratospheric aerosol injection has been proposed as a policy response to warming from human emissions of greenhouse gases, but it may produce unequal regional impacts. We present a simple, intuitive risk-based framework for classifying these impacts according to whether geoengineering increases or decreases the risk of substantial climate change, with further classification by the level of existing risk from climate change from increasing carbon dioxide concentrations. This framework is applied to two climate model simulations of geoengineering counterbalancing the surface warming produced by a quadrupling of carbon dioxide concentrations, with one using a layer of sulphate aerosol in the lower stratosphere, and the other a reduction in total solar irradiance. The solar dimming model simulation shows less regional inequality of impacts compared with the aerosol geoengineering simulation. In the solar dimming simulation, 10% of the Earth's surface area, containing 10% of its population and 11% of its gross domestic product, experiences greater risk of substantial precipitation changes under geoengineering than under enhanced carbon dioxide concentrations. In the aerosol geoengineering simulation the increased risk of substantial precipitation change is experienced by 42% of Earth's surface area, containing 36% of its population and 60% of its gross domestic product.

## Introduction

Geoengineering by injection of aerosol into the stratosphere has been proposed as a possible countermeasure to climate warming driven by human emissions of greenhouse gases [1]. Sulphate aerosol is most commonly proposed, though other aerosol types could also be used [2]–[5]. Climate model simulations have suggested that stratospheric aerosol geoengineering can be used to effectively reduce Earth's global mean surface temperature [6], [7], but that it is not possible to simultaneously minimise changes in both surface temperature and precipitation [8]–[10].

The impacts of geoengineering are also unlikely to be regionally uniform [9]–[12]. Therefore some regions may benefit more from geoengineering than others, and there may potentially be some regions for which the impacts of geoengineering are more undesirable than those of unabated CO_2_-driven climate change. In addition, individual regions may have different preferences on the amount of cooling required [9], [10]. Therefore, even if there is a universal global benefit associated with geoengineering, inequality of benefits could still lead to conflict [13]. There may also be diverse views on the appropriate goal for geoengineering: for example, should geoengineering be optimised to protect the most people from climate changes, or to protect key global economic regions? [10] The level of inequality in impacts will also depend on the chosen goal [11].

Another possible geoengineering scheme is the placement of reflectors in space to reduce the incoming solar radiation. This technique can be simulated in climate models by reducing the amount of solar radiation reaching the top of Earth's atmosphere, termed the total solar irradiance. For practical reasons some climate model simulations adopt this approach to represent stratospheric aerosol geoengineering. Such simulations include the ‘G1’ scenario of the Geoengineering Model Intercomparison Project (GeoMIP) [14]. However, the spatial distribution of a reduction in solar irradiance on the global radiation balance may not be the same as a geoengineering aerosol layer. In addition, solar dimming does not represent the radiative effects of aerosol on the stratosphere [2], which may lead to different impacts [15]. Therefore it is important to assess the extent to which solar dimming experiments are useful in quantifying the regional impacts of solar radiation.

Geoengineering can be thought of as an approach to managing climate risk [16]. The success of a geoengineering scheme could be described by the extent to which it reduces the risk of significant climate changes (though the definition of ‘significant’ is subjective).

In the field of epidemiology a risk-based approach is often adopted to disease treatment trials [17]. A particular view of geoengineering could be analogous to this approach. In this analogy, geoengineering is a treatment for the symptoms of a disease (elevated atmospheric CO_2_ concentrations). Note that sunlight reflection geoengineering treats only the symptoms of the disease, which are in this case climatic changes including global-mean surface warming, rather than the disease itself. The success of the treatment is judged by the extent to which it reduces the risk of the planet experiencing the symptoms. This analogy has clear relevance for geoengineering policy, in that the level of risk reduction could be used to justify or prohibit the deployment of the ‘treatment’.

In this paper we present a simple risk-based framework for the assessment of the regional impacts of stratospheric aerosol geoengineering. We test the framework using climate model simulations of geoengineering represented by sulphate aerosol and by solar dimming. We find that when geoengineering is represented by solar dimming, the risks associated with geoengineering are underestimated compared to the sulphate aerosol simulations.

## Methods

### Climate model simulations

The University of Reading Intermediate General Circulation Model (IGCM) [18] is used to simulate high-CO_2_ and geoengineered climates. The model is coupled to a mixed-layer ‘slab’ ocean 100 m in depth. Using a ‘slab’ ocean allows the model to equilibrate rapidly to perturbations. Each simulation is 80 years in length and the final 65 years are analysed (thus allowing 15 years for the climate to equilibrate to radiative forcings). A ‘slab’ ocean, being static, needs calibration to represent the effects of the ocean circulation on heat transport. Ocean heat fluxes are calculated from the surface energy imbalance when the IGCM is run with sea surface temperatures fixed with a monthly climatology from the ERA-40 reanalysis [19]. The model is run with a spectral resolution of T42 (triangular truncation of wavenumbers greater than 42 – a horizontal resolution of approximately 2.7 degrees) with 35 vertical layers up to 0.1 hPa.

The climate model simulations are shown in Table 1. The ‘Control’ simulation represents a 20^th^ Century climate. The ‘4CO_2_’ simulation has quadrupled CO_2_ concentrations, representing an undesirable climate state in which substantial greenhouse gas emissions have produced global-mean surface warming (4.20 K – see Table 1). Two geoengineering simulations are used. In ‘4CO_2_ + Sulphate’ the quadrupling of CO_2_ is counterbalanced by prescribing a time-invariant zonally uniform layer of sulphate aerosol in the lower stratosphere (described in Text S1 and illustrated in Figure S1). In ‘4CO_2_ + Solar’ the quadrupling of CO_2_ is counterbalanced using a reduction in total solar irradiance, after the GeoMIP protocol [14].

**Table 1:** Climate model simulations.

Simulation name	CO_2_ concentration (ppmv)	Geoengineering	Global-mean surface temperature change (K)	Global-mean precipitation change (mm/day)
Control	355	-	0	0
4CO_2_	1420	-	4.20	0.20
4CO_2_ + Sulphate	1420	Prescribed sulphate aerosol layer	−0.28	−0.25
4CO_2_ + Solar	1420	3.4% reduction in total solar irradiance	0.10	−0.10

The sulphate aerosol in ‘4CO_2_ + Sulphate’ interacts with both shortwave and longwave radiation. Representation of the effects of aerosol on the full spectrum of electromagnetic radiation is important because, though sulphate is primarily scattering at visible wavelengths, it produces non-negligible absorption at longer wavelengths [2]. It is assumed to have a lognormal size distribution with a median radius of 0.1 µm and a geometric standard deviation of 2.0, based on previous studies of stratospheric aerosol geoengineering using aerosol microphysical models [20].

### Risk analysis framework

We present a novel framework for the assessment of regional climate risk in high-CO_2_ and geoengineered climates, based on the probability of exceedance of a target climate threshold at a particular location in any given year. A threshold could be chosen for any climate variable of interest. In this paper we simply consider annual-mean temperature and precipitation; in the Discussion we address the possibility of including other variables.

The risk ratio is defined as:
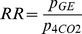



Where 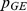 is the probability of exceedance of the threshold in the geoengineered climate (‘4CO_2_ + Sulphate’ or ‘4CO_2_ + Solar’) and 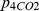 is the probability of exceedance of the threshold in the ‘4CO_2_’ climate. Therefore if RR>1, geoengineering increases the risk of exceeding the given climate change threshold relative to the un-geoengineered ‘4CO_2_’ climate.

For illustrative purposes, in this paper the threshold is taken to be when the annual-mean climate state in a perturbed climate differs from the control climatology by greater than 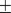
1 standard deviation (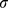) of the interannual variability. We assume that climate damages associated with this change do not depend on the sign of this change, i.e. that a negative change in a given climate variable is as undesirable as a positive change [10]. This is not necessarily the most appropriate approach for considering the impacts of climate change, since some regions may be more sensitive to climate changes of a certain sign. The possibility of incorporating sign-sensitivity into the framework is addressed in the Discussion.

The annual probability of exceedance is calculated at each spatial point as the fraction of years exceeding the 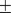
1 standard deviation threshold.

A risk ratio greater than 1 has two possible implications: either that geoengineering enhances the magnitude of climate change caused by a quadrupling of CO_2_, or that the geoengineering produces substantial climate changes where there were none under a quadrupling of CO_2_. In the former case geoengineering has exacerbated the existing climate risk caused by CO_2_, whereas in the latter geoengineering has introduced climate risk in a region where there was none under a quadrupling of CO_2_.

A risk ratio of less than 1 also has two possible implications: that geoengineering has reduced the existing climate risk of CO_2_, or that geoengineering has reduced climate risk in a region which was not at risk of substantial climate change under a quadrupling of CO_2_ anyway.

**Figure 1:**
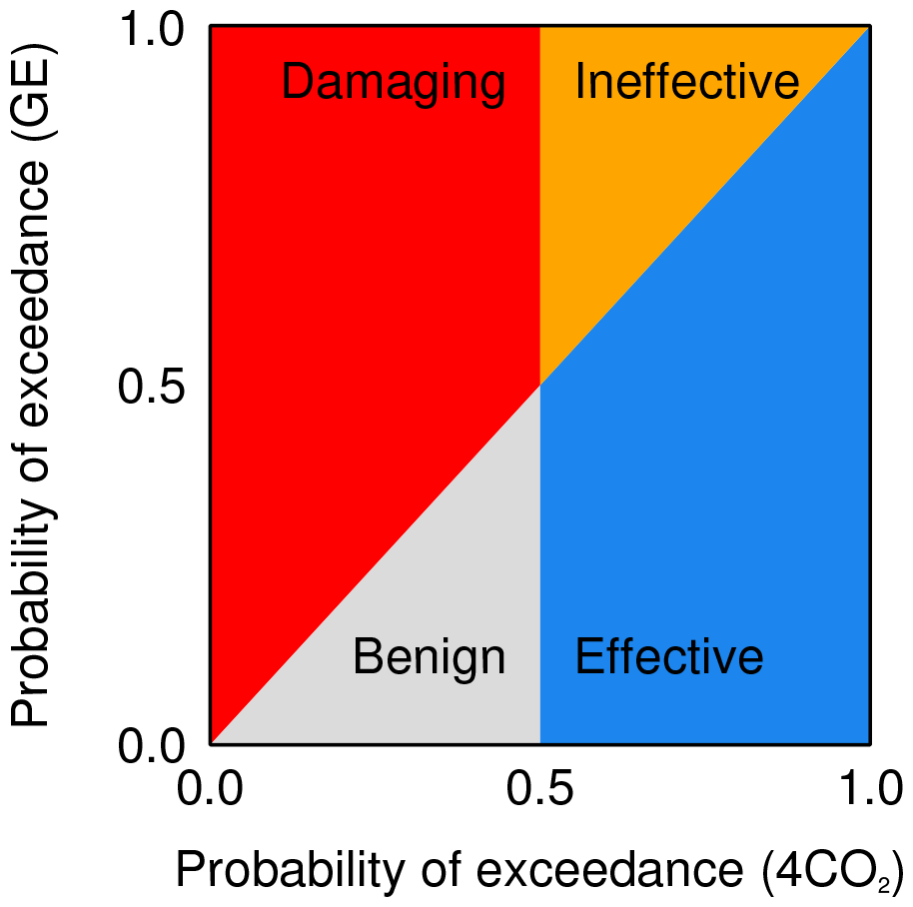
Matrix for classifying impacts of geoengineering (GE) by comparing its effect with a quadrupled-CO_2_ scenario.

Taken together there are four possible outcomes, expressed in Figure 1 as regions on a scatter plot of the probability of exceedance for 4CO_2_ against the probability of exceedance for geoengineering. We adopt the following definitions of the outcomes:

**Damaging**. Risk increased in areas not at risk. In areas where a 1
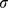
change was *less* likely than not (i.e. 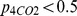) under 4CO_2_, geoengineering *increases* the likelihood of a 1
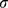
change.
**Ineffective**. Risk increased in at-risk areas. In areas where a 1
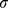
change was *more* likely than not (i.e. 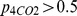) under 4CO_2_, geoengineering *increases* the likelihood of a 1
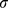
change.
**Benign**. Risk reduced in areas not at risk. In areas where a 1
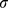
change was *less* likely than not (i.e. 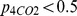) under 4CO_2_, geoengineering *decreases* the likelihood of a 1
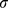
change.
**Effective**. Risk reduced in at-risk areas. In areas where a 1
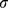
change was *more* likely than not (i.e. 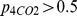) under 4CO_2_, geoengineering *decreases* the likelihood of a 1
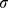
change.

Thus areas which experience ‘damaging’ and ‘ineffective’ changes have a risk ratio of greater than 1, and areas which experience ‘benign’ and ‘effective’ changes have a ratio ratio of less than 1. The choice of the change and likelihood thresholds depends on the application (addressed in the Discussion). However, in order to illustrate the framework clearly and generally, we adopt the simple approach outlined above.

Some apparent climate change signals may in fact be due to natural variability. This could lead to misclassification of variability-driven signals as a consequence of geoengineering. This problem occurs when the difference between perturbed (geoengineered or 4CO_2_) and control climates is small. When the probability of exceedance of the threshold is small for *both 4CO_2_ and geoengineering* (i.e. the region close to the origin in Figure 1) no conclusion can be drawn because the distinction between ‘damaging’ and ‘benign’ will be dominated by natural variability rather than forced climate changes. We therefore exclude from the analysis those regions where the response (compared to the control simulation) is not statistically significant at the 95% level in *either* the 4CO_2_ or geoengineering simulations. This step ensures that it is likely that the regions admitted to the analysis are comparing real forced signals rather than changes associated with natural variability. Figure S2 shows, within the framework presented in Figure 1, those regions where the response to geoengineering is unclassifiable according to this criterion.

This framework assigns simple terms to the effects of geoengineering on climate. Each term also gives information on climate risk. If geoengineering is ‘damaging’, risk has been *introduced* where there was none before. We use the term ‘damaging’ since this outcome implies climate change from geoengineering in areas which might not be prepared to adapt to climate change produced by greenhouse gases, so resilience may be lower. If geoengineering is ‘ineffective’, risk has been increased (or sustained) in areas which were at risk from substantial climate change under the high-CO_2_ scenario. If geoengineering is ‘benign’, risk has been decreased, but the response in the high-CO_2_ is small enough that there was little risk to begin with. If geoengineering is ‘effective’, risk has been decreased where there is risk of severe climate change from CO_2_. This choice of terms is subjective and applications of this framework to specific climate impacts may be better suited to a different set of terms.

‘Ineffective’ geoengineering does not necessarily imply that geoengineering has little effect on the climate variable of interest. It simply means that geoengineering has not reduced the risk of severe climate change. The climate response in the 4CO_2_+Sulphate and 4CO_2_+Solar simulations will be a combination of the responses to a quadrupling of CO_2_ and geoengineering. If geoengineering is classified as ‘ineffective’, this implies either:

The climate response to geoengineering is small and the response in the geoengineering simulations is dominated by the 4CO_2_ component.
The climate response to geoengineering is large (and potentially of the opposite sign to 4CO_2_), but does not return the local climate to the control baseline.

Thus it is possible for the climate response to geoengineering to be classified as ‘ineffective’, while at the same time being very different to the response to a quadrupling of CO_2_ alone.

## Results

**Figure 2:**
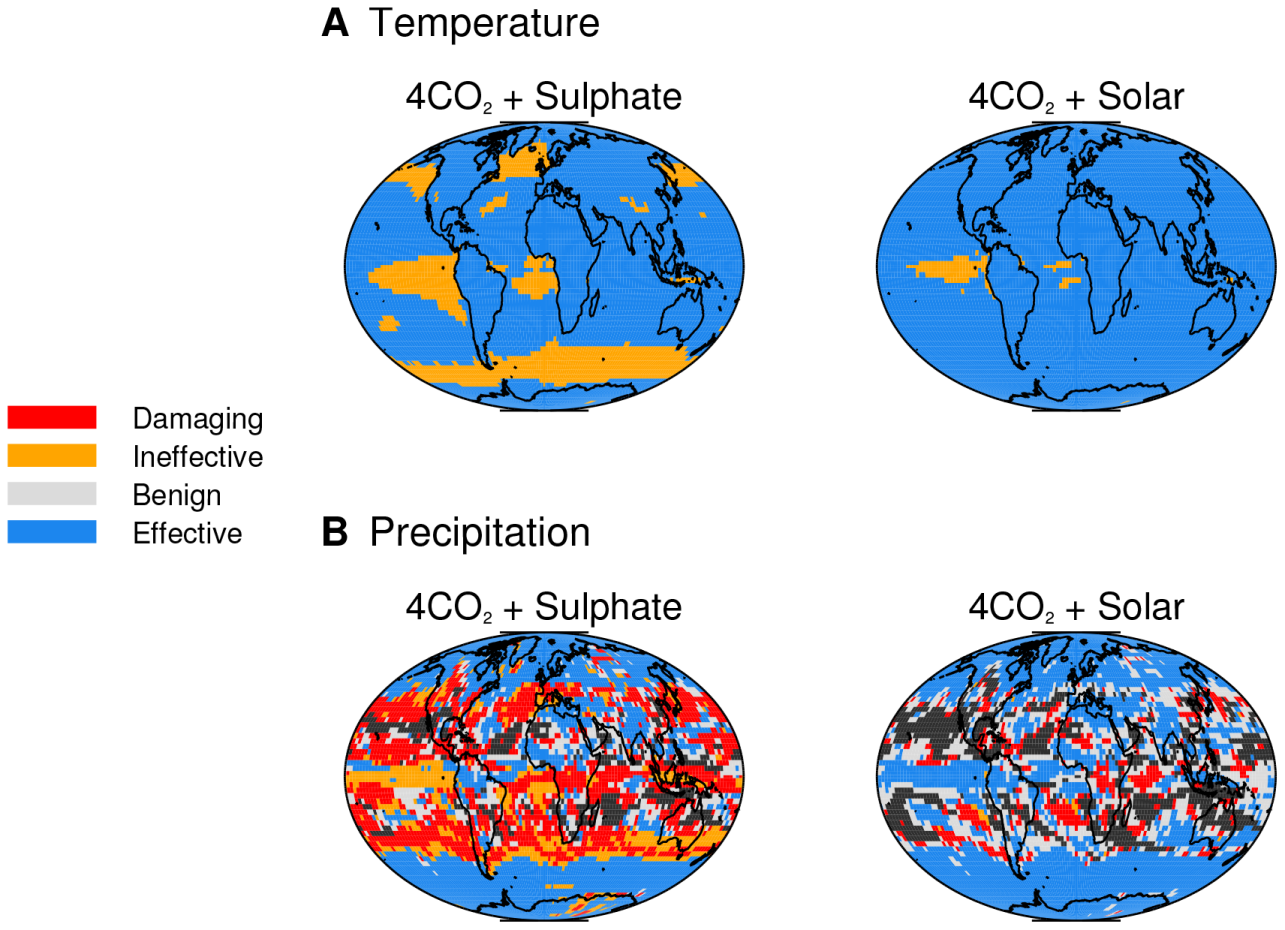
Maps of outcomes of geoengineering. The risk-based framework (illustrated in Figure 1) is used to classify outcomes for (a) annual-mean climatological surface temperature and (b) annual-mean climatological precipitation. Black shading denotes regions where neither the response to 4CO_2_ or geoengineering are statistically significant at the 95% level (making it impossible to accurately classify the effectiveness of geoengineering).

Here we present illustrative results of the effects of geoengineering on climate risk using the simple framework described above together with the climate model simulations of geoengineered and quadrupled-CO_2_ climates. In this framework, geoengineering is broadly effective at counterbalancing regional changes in annual-mean surface temperature (Figure 2A). This is to be expected since minimisation of global-mean surface temperature change was an explicit goal of the climate model simulation. A greater area is classified as ‘ineffective’ in the ‘4CO_2_ + Sulphate’ simulation, indicating more regional inhomogeneity in this simulation than ‘4CO_2_ + Solar’. However, nowhere does geoengineering increase the risk of 1
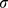
changes in surface temperature where there was none before (which, under our framework, would be classified as ‘damaging’). Since quadrupling CO_2_ concentrations produces substantial warming everywhere, none of the spatial points are masked out as statistically insignificant (recall that small changes, when neither the responses to CO_2_ or geoengineering are statistically significant, cannot be classified).

Consistent with previous climate modelling studies [8]–[10], our climate model simulations show that geoengineering to minimise global-mean surface temperature change cannot minimise global-mean precipitation change (Table 1). This is a robust result of the different vertical profiles of the radiative forcings of CO_2_ and shortwave flux reductions [8], [12], and is seen in both geoengineering simulations. However, Figure 2B shows that the two geoengineering simulations have different effects on regional climate risk of annual-mean precipitation change. In the Equatorial and subtropical regions sulphate aerosol geoengineering mostly increases climate risk from precipitation change, whereas the area over which solar dimming increases risk is much smaller. A greater area remains unclassified in the 4CO_2_+Solar case than the 4CO_2_+Sulphate case, indicating the magnitude of the regional precipitation response is generally smaller in 4CO_2_+Solar.

Most notably, however, a much larger area of the Earth is ‘damaged’ by geoengineering in the sulphate case than the solar dimming case (red shading in Figure 2). This indicates that geoengineering introduces risk of substantial climate changes in regions where there was no risk under a quadrupling of CO_2_.

In both simulations geoengineering is effective in high-latitude regions, indicating geoengineering has reduced the climate risk of precipitation changes from a quadrupling of CO_2_.

We now apply this regional analysis to potential policy-relevant metrics of sensitivity to climate change. We calculate the fraction of the global area affected by dangerous, ineffective, benign and effective geoengineering and compare this with the fraction of global population and the total GDP of the affected regions. Population data for the year 2000 are obtained from the Gridded Population of the World version 3 dataset [21] and GDP data for the year 2005 are obtained from the G-Econ dataset [22].

**Figure 3:**
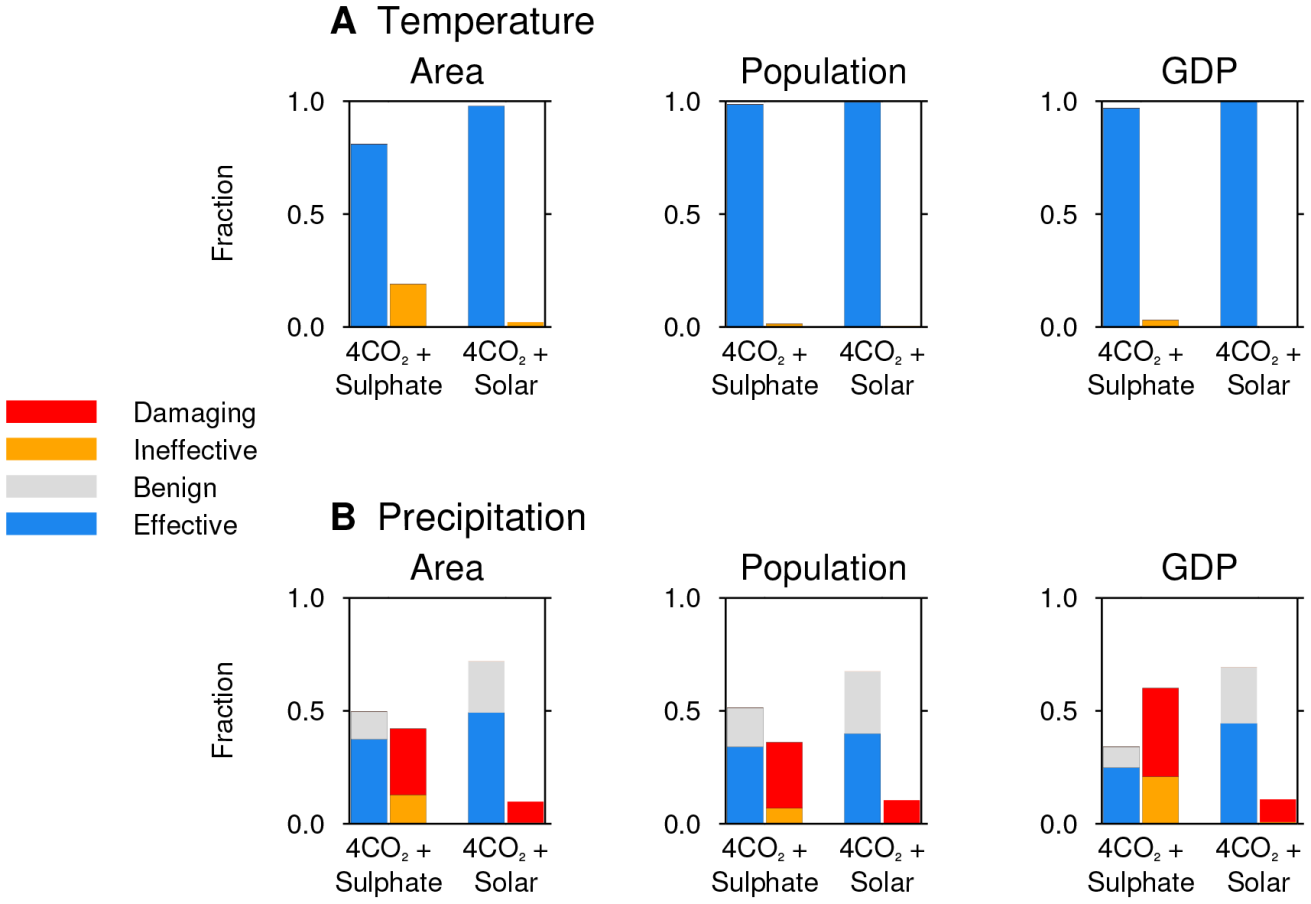
Fraction of global area, population and GDP affected by different outcomes of geoengineering. Each climate model simulation has a pair of bars. The left-hand bar shows the ‘benign’ and ‘effective’ outcomes, i.e. where geoengineering reduces risk. The right-hand bar shows the ‘damaging’ and ‘ineffective’ outcomes, i.e. where geoengineering increases risk. Regions where neither the response to 4CO_2_ or geoengineering are statistically significant at the 95% level are neglected, so the bars do not sum to 1.0.

While some of the Earth experiences ineffective reduction in risk of surface temperature change in ‘4CO_2_ + Sulphate’ (Figure 2A), all of these regions are oceanic. Consequently, nearly all of the Earth's population and GDP escape this increase in climate risk (Figure 3A), as is the case for the ‘4CO_2_ + Solar’ simulations.

Sulphate geoengineering, however, approximately doubles the global area experiencing increased risk of substantial precipitation change when compared to the solar dimming simulation (Figure 3B). In ‘4CO_2_ + Sulphate’ nearly 50% of the Earth's surface area experiences this increase in risk.

A majority of the world's population experiences a reduction in the climate risk from precipitation change in both simulations. This indicates that geoengineering reduces risk for most of the world's population, but this majority is much smaller in the ‘4CO_2_ + Sulphate’ case.

This distinction between sulphate and solar dimming geoengineering becomes greater when the contribution of different regions to global GDP is considered. The right-hand panel of Figure 3b shows that around 60% of the world's economic output (as measured by GDP) resides in regions in which sulphate geoengineering increases the risk of precipitation changes, and approximately 40% in regions that are damaged. In the solar dimming simulation the GDP fraction in regions with increased risk of precipitation change is approximately 15%.

## Discussion

We have presented a simple, intuitive framework for describing the regional climate impacts of geoengineering. In this framework it is assumed the goal of geoengineering is the reduction of the risk of exceeding a given climate threshold in a given year, and that the sign of the change is not important. In this framework geoengineering may be considered successful if this risk is reduced.

Using this framework, we show that there is substantial regional variation in effectiveness of geoengineering in mitigating precipitation changes (in addition to global-mean changes), and that these impacts and their regional variations are underestimated when geoengineering is represented by a simple reduction in total solar irradiance rather than using a stratospheric aerosol layer. These results suggest caution is required when interpreting climate model experiments which represent geoengineering using solar dimming, and that correct representation of the stratospheric aerosol layer is required to correctly characterise the regional impacts.

Since the risk metrics presented here are based on results from a single climate model of intermediate complexity we do not suggest the specific regional impacts identified in this paper are a good measure of the potential real-world impacts of stratospheric aerosol geoengineering. In addition, the representation of the stratospheric aerosol used here is highly idealised, since the aerosol is not allowed to interact with the atmospheric circulation. Other climate model simulations of geoengineering might produce different results, but the simple risk framework presented here, with four clearly-defined outcomes, may be helpful in comparing simulations and assessing the robustness of regional impacts. It may also prove useful in modelling studies attempting to optimise the deployment of geoengineering to minimise negative impacts.

The global area affected by different outcomes of geoengineering in this framework is very sensitive to the chosen definition of ‘substantial change’. In this paper, for illustrative purposes, we have assumed the goal of geoengineering to be a reduction in the risk of experiencing a year in which the mean surface temperature or precipitation is outside 1 standard deviation of the current interannual variability. Figures S3 and S4 show results corresponding to Figures 2 and 3 in which the chosen threshold is 2 standard deviations. In this case a large surface area experiences changes in which geoengineering is considered ‘benign’, but this is because the quadrupled-CO_2_ simulation rarely breaches the threshold (due to large interannual variability in precipitation), and so is rarely classified as damaging. Therefore, in this framework the conclusions of the analysis depend strongly on the initial choice of threshold, and this threshold should be carefully selected.

The framework can however be used flexibly, with the goal of geoengineering and the threshold over which climatic changes are damaging chosen according to policy requirements. In addition, depending on the application, a time-resolution of greater than 1 year may also be appropriate. For example, depending on a particular region's sensitivity, droughts may occur when there is a sustained rainfall deficit over smaller timescales [23]. The framework could also be used to assess the impact of geoengineering on climate risk depending on the season. For example, food production regions would be more sensitive to climatic changes during the growing season.

Since global-mean precipitation is reduced in geoengineering simulations, most of the regional changes are also reductions [8], [10], [12]. The regional precipitation response to carbon dioxide increase is, on the other hand, mixed. Therefore, sometimes geoengineering and carbon dioxide can act to drive similar magnitudes of climate change but of different signs. Depending on the application a weighting could be applied to the risk ratio results to reflect the potential asymmetry in the damage inflicted by increases and decreases in climate variables. Such a weighting could be applied on a regional basis to account for different regional sensitivities to climatic changes.

Multiple variables could also be incorporated into the analysis, potentially by introducing a ‘loss function’ at each model grid point, representing that region's sensitivity to changes in temperature, precipitation or other climate variables. In the example of food production, changes in soil moisture would be relevant. A loss function would need to describe whether a particular region's agricultural productivity was limited by water supply or by other conditions.

The framework presented in this paper could also be used as part of a cost-benefit study of geoengineering. However, a cost-benefit approach introduces further uncertainties because the conversion of changes in physical variables as simulated by a climate model into meaningful monetary costs and benefits is not straightforward. In addition, there will almost certainly be unknown climate risks associated with any kind of climate change (from greenhouse gases or geoengineering) that cannot be simulated by climate models. We therefore propose this framework primarily as a way to present climate model results in a simple and meaningful fashion, keeping in mind these models' capabilities. The framework allows comparison between results from different climate models as well as the outcomes from different levels of geoengineering. Simple metrics such as the population fraction experiencing increased climate risks could be used to calculate the optimal level of geoengineering when multiple climate variables are taken into account [10] (e.g. simultaneously minimising changes in temperature and precipitation, taking into account different regions' sensitivities).To return to the analogy of disease treatment introduced at the beginning of this paper, we see that geoengineering can be used as a treatment to alleviate the symptoms of elevated atmospheric CO_2_ concentrations. However, the treatment itself carries risks, and substantial parts of the world (whether measured by area, population or economic activity) experience greater risk when the geoengineering treatment is applied than when the effects of CO_2_ on their climate are unabated.

## Supporting Information

Figure S1 Zonal-mean aerosol mass mixing ratio. The aerosol distribution is used in ‘4CO_2_ + Sulphate’. Units are 10^−6^ kg/kg.
(TIF)

Figure S2 Scatter plot of exceedance probabilities in 4CO_2_ and geoengineering (GE) scenarios. Each point represents one climate model grid box. The probabilities of exceedence are calculated as the fraction of years in the climate model simulations exceeding 1 standard deviation of the interannual variability. Shaded regions indicate the classification of the responses according to the framework described in the main text. Black crosses indicate spatial points at which the climatological response is not statistically significant at the 95% level in either the 4CO_2_ or GE scenario.
(TIF)

Figure S3 Maps of outcomes of geoengineering using a 2σ threshold for CO_2_ changes becoming substantial. The risk-based framework (illustrated in Figure 1) is used to classify outcomes for (a) annual-mean climatological surface temperature and (b) annual-mean climatological precipitation.
(TIF)

Figure S4 Fraction of global area, population and GDP affected by different outcomes of geoengineering, using a 2σ threshold for CO_2_ changes becoming substantial. Each climate model simulation has a pair of bars. The left-hand bar shows the ‘benign’ and ‘effective’ outcomes, i.e. where geoengineering reduces risk. The right-hand bar shows the ‘damaging’ and ‘ineffective’ outcomes, i.e. where geoengineering increases risk.
(TIF)

Text S1 (DOCX)
