# Supplementary figures and images for: A Risk-Based Framework for Assessing the Effectiveness of Stratospheric Aerosol Geoengineering
Source: PLoS One. 2014 Feb 12;9(2):e88849. doi: 10.1371/journal.pone.0088849 (PMC3923064; doi:10.1371/journal.pone.0088849)

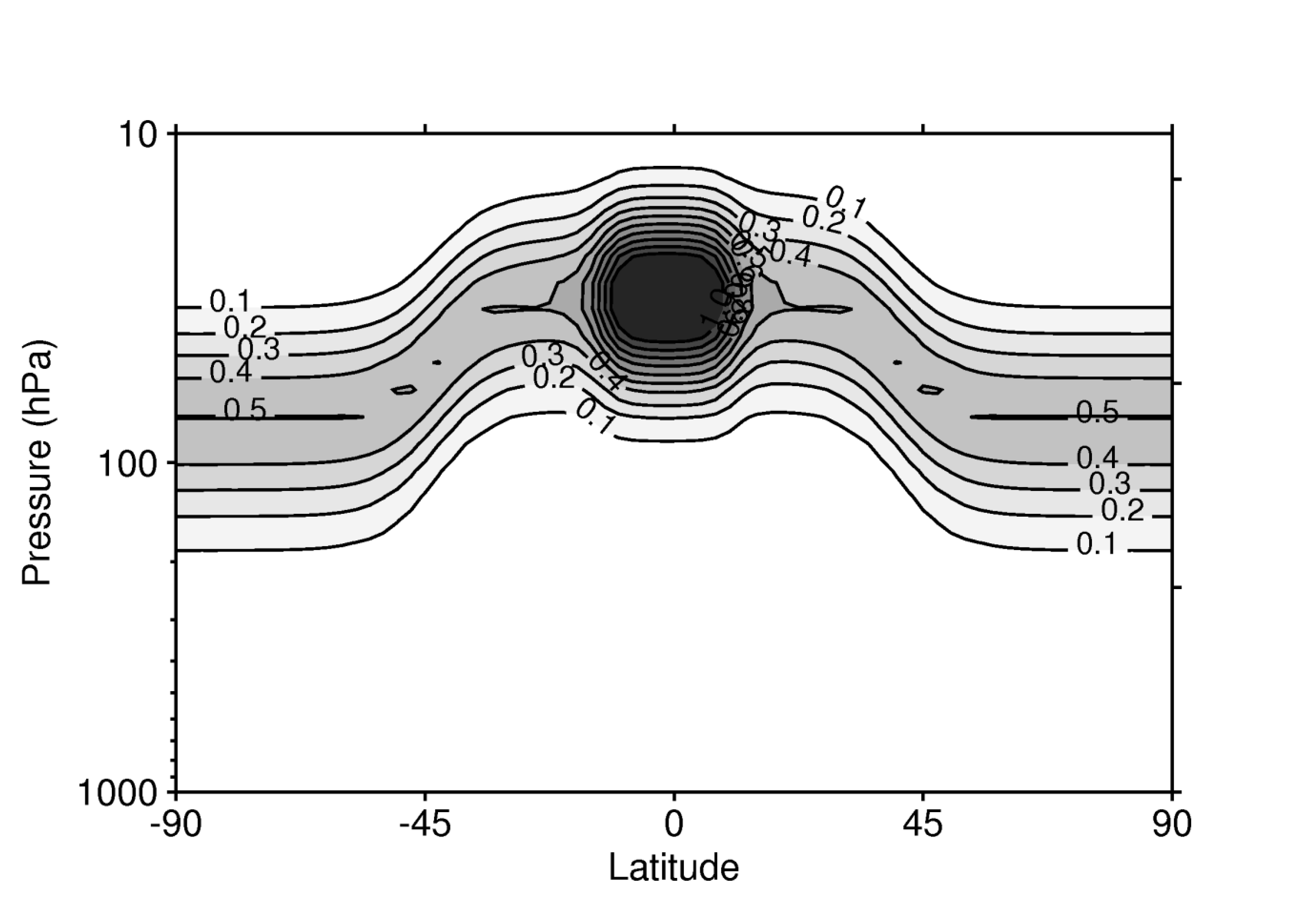

Supplement: Figure S1 — Zonal-mean aerosol mass mixing ratio. The aerosol distribution is used in ‘4CO2 + Sulphate’. Units are 10−6 kg/kg. (TIF) [file pone.0088849.s001.tif]

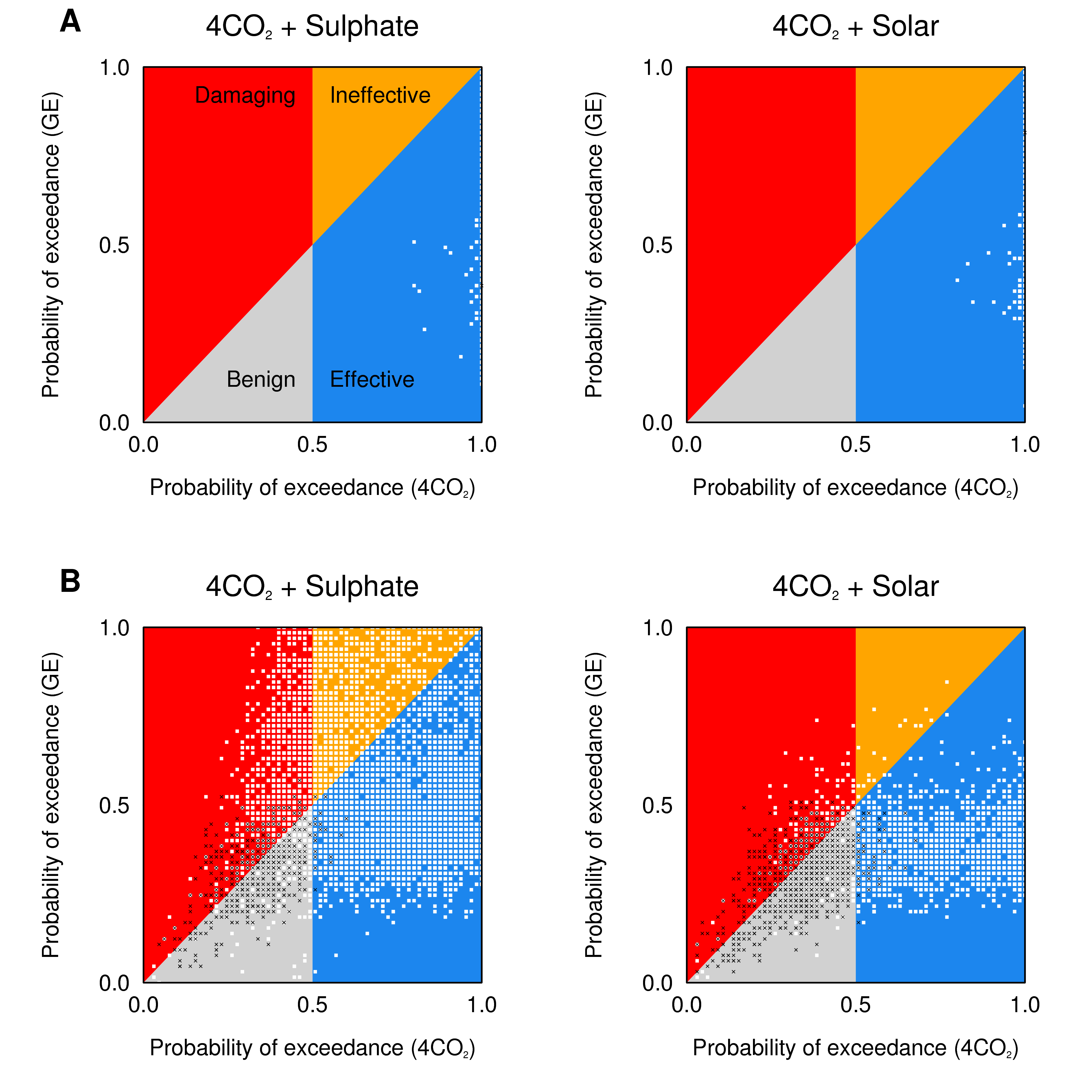

Supplement: Figure S2 — Scatter plot of exceedance probabilities in 4CO2 and geoengineering (GE) scenarios. Each point represents one climate model grid box. The probabilities of exceedence are calculated as the fraction of years in the climate model simulations exceeding 1 standard deviation of the interannual variability. Shaded regions indicate the classification of the responses according to the framework described in the main text. Black crosses indicate spatial points at which the climatological response is not statistically significant at the 95% level in either the 4CO2 or GE scenario. (TIF) [file pone.0088849.s002.tif]

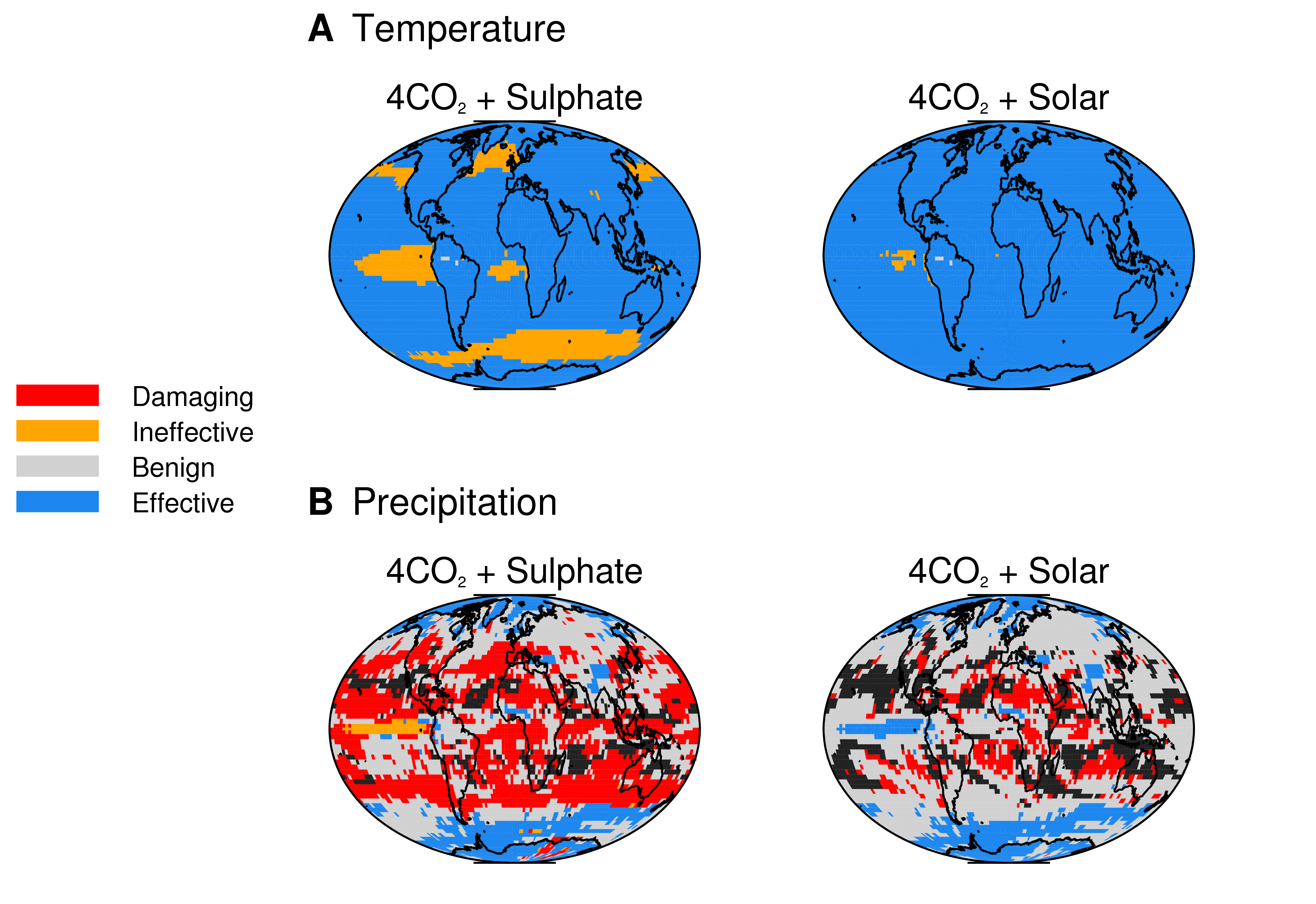

Supplement: Figure S3 — Maps of outcomes of geoengineering using a 2σ threshold for CO2 changes becoming substantial. The risk-based framework (illustrated in Figure 1) is used to classify outcomes for (a) annual-mean climatological surface temperature and (b) annual-mean climatological precipitation. (TIF) [file pone.0088849.s003.tif]

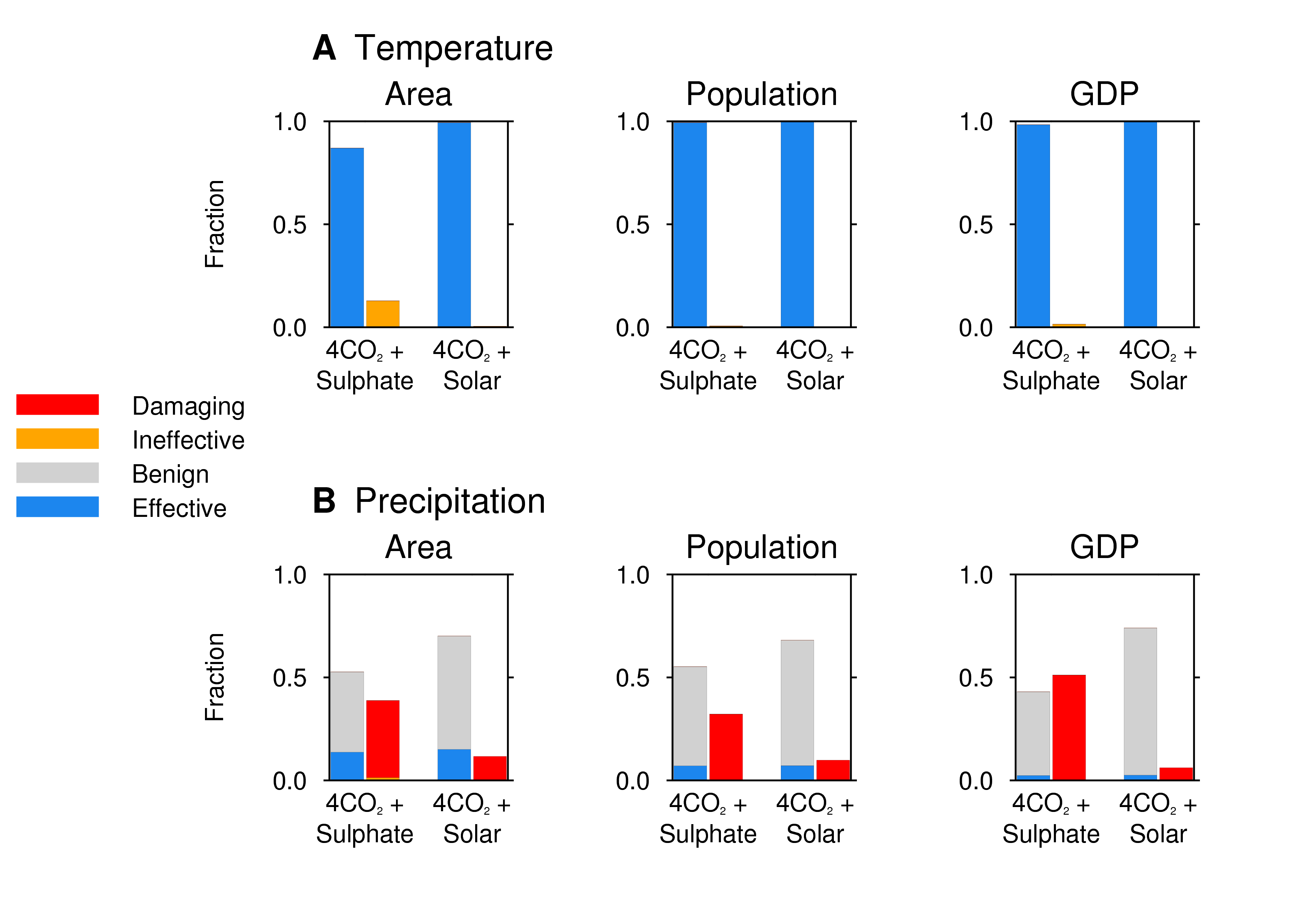

Supplement: Figure S4 — Fraction of global area, population and GDP affected by different outcomes of geoengineering, using a 2σ threshold for CO2 changes becoming substantial. Each climate model simulation has a pair of bars. The left-hand bar shows the ‘benign’ and ‘effective’ outcomes, i.e. where geoengineering reduces risk. The right-hand bar shows the ‘damaging’ and ‘ineffective’ outcomes, i.e. where geoengineering increases risk. (TIF) [file pone.0088849.s004.tif]
