# Supplementary material for: A Risk-Based Framework for Assessing the Effectiveness of Stratospheric Aerosol Geoengineering
Source: PLoS One. 2014 Feb 12;9(2):e88849. doi: 10.1371/journal.pone.0088849 (PMC3923064; doi:10.1371/journal.pone.0088849)
Supplement: Text S1 — (DOCX) [file pone.0088849.s005.docx]

**A risk-based framework for assessing the effectiveness of stratospheric aerosol geoengineering**

**Angus J Ferraro, Andrew J Charlton-Perez, Eleanor J Highwood**

Department of Meteorology, University of Reading, Reading, UK

**SUPPORTING INFORMATION**

**1. Aerosol layer representation**

Stratospheric aerosol geoengineering is modelled in two ways in this study: a reduction in total solar irradiance at the top of the atmosphere, and a prescribed, time-invariant, zonally symmetric distribution of stratospheric sulphate aerosol. In choosing a form for this distribution, we assume there exists some injection scheme which can maintain an aerosol layer in a steady state by injection of sulphate aerosol or a precursor gas like SO_2_. The spatial distribution of sulphate fulfils two criteria:

1. Peak mass mixing ratio at the injection point. This is because some aerosol mass is removed from the stratosphere by gravitational settling before it is transported polewards by the Brewer-Dobson circulation (as demonstrated in simulations with aerosol microphysical models [1], [2]). Therefore a constant flux of aerosol mass into the Tropics and a constant flux of aerosol mass polewards in the Brewer-Dobson circulation actually leads to a non-uniform aerosol distribution, due to depletion by the additional flux of aerosol mass into the troposphere by gravitational settling (once in the troposphere it is removed on timescales of weeks by wet deposition).
2. Aerosol at higher altitude in the Tropics and lower altitude at higher latitudes, reflecting the influence of the Brewer-Dobson Circulation (which has rising motion in the Tropics and transport to the poles).

We use an analytical function (consisting of a tanh function in the horizontal and a Gaussian in the vertical) to represent these characteristics. The distribution of aerosol mass mixing ratio *M* is:

$M\left( \left| \varphi\right|,z \right)=f\left( \left| \varphi\right| \right)g\left( z \right)$ (1)

where $\varphi$ is latitude (in degrees) and *z* is altitude (in km). The two functions representing variations in latitude and altitude are:

$g\left( z \right)=Aexp\left( \frac{-(z-B\left( \left| \varphi\right| \right)^{2}}{2C^{2}} \right)$ (2)

$f\left( \left| \varphi\right| \right)=\mathrm{Dtanh} \left( E\left| \varphi\right|-F \right)+G$ (3)

These functions have the following parameters:

$A=2.17\times{10}^{-6}, B\left( \left| \varphi\right| \right)=Htanh\left( I\left| \varphi\right|-J \right)+K), C=3.0, D=0.5, E=0.25, F=20, G=1.11, H=3.0, I=0.1, J=5, K=19.5$

The distribution is shown in Figure S1.

**2. Sensitivity of classification of response to choice of threshold**

The classification of the response to climate changes depends on the choice of threshold for which the changes are termed substantial. In the main article, the change is defined as substantial when the probability in increased of a single year exceeding 1 standard deviation (σ) of the interannual variability. Here we present results for when the threshold is 2σ (Figures S3 and S4).

When a more stringent threshold is used, a greater area experiences ‘benign’ geoengineering. This is because a smaller area of the planet experiences 2σ changes than 1σ changes in precipitation following a quadrupling of CO_2_. Thus, under this particular set of assumptions, geoengineering might not be considered worthwhile simply because the changes in precipitation patterns associated with CO_2_ are not that large. However, a 2σ change in annual-mean precipitation is very substantial due to the large interannual variability. These results illustrate the importance within this framework of selecting a threshold of substantial climate change which is physically-relevant and/or policy-relevant. Otherwise, the impacts may not be correctly classified.

**References**

[1] Niemeier U, Schmidt H, Timmreck C (2011) The dependency of geoengineered sulfate aerosol on the emission strategy. Atmos Sci Lett 12: 189–194 doi:10.1002/asl.304.

[2] Heckendorn P, Weisenstein DK, Fueglistaler S, Luo BP, Rozanov E V., et al. (2009) The impact of geoengineering aerosols on stratospheric temperature and ozone. Environ Res Lett 4: 045108 doi: 10.1088/1748-9326/4/4/045108.
